# Supplementary material for: Copy number losses of oncogenes and gains of tumor suppressor genes generate common driver mutations
Source: Nat Commun. 2024 Jul 20;15:6139. doi: 10.1038/s41467-024-50552-1 (PMC11271286; doi:10.1038/s41467-024-50552-1)
Supplement: Supplementary file 1 — Supplementary Information [file 41467_2024_50552_MOESM1_ESM.pdf]

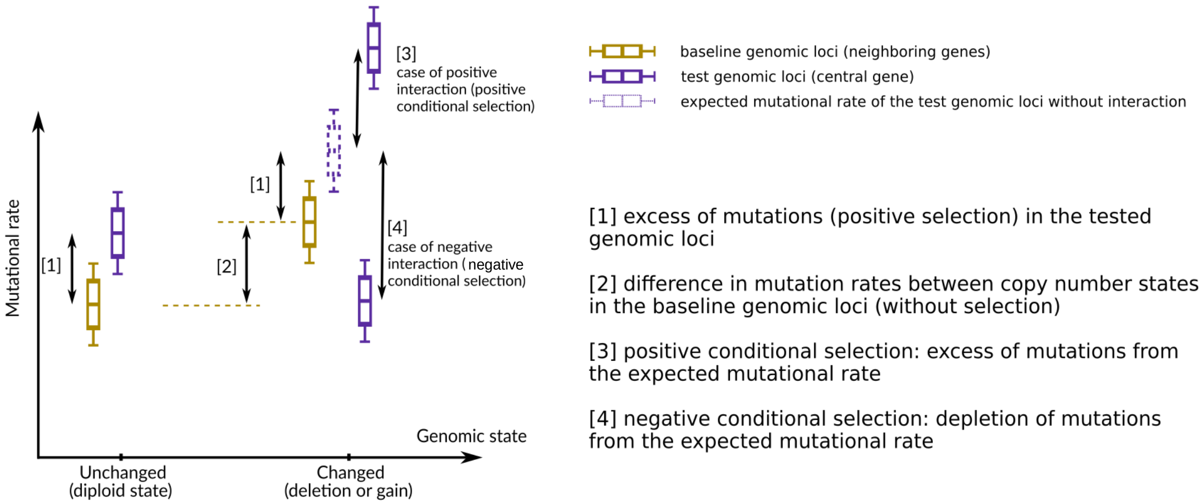

Supplementary Figure S1. Selection model used to estimate the conditional selection upon the change of genomic copy number state. This model evaluates how genomic copy number variations influence the mutational rate (as indicated in [2], with a baseline established from neighboring genes) and selection pressure ([1], determined by the difference in mutation rates between the test and baseline genomic locations). The anticipated mutation rate within the test loci in changed copy number state is illustrated using a dotted boxplot. This representation is based on the assumption that the effect of copy number variations and the genomic loci state on the mutation rate are independent. Any deviation between the expected and the actual mutation rates observed in the test loci (either an increase [3] or a decrease [4]) represent the impact of conditional selection.

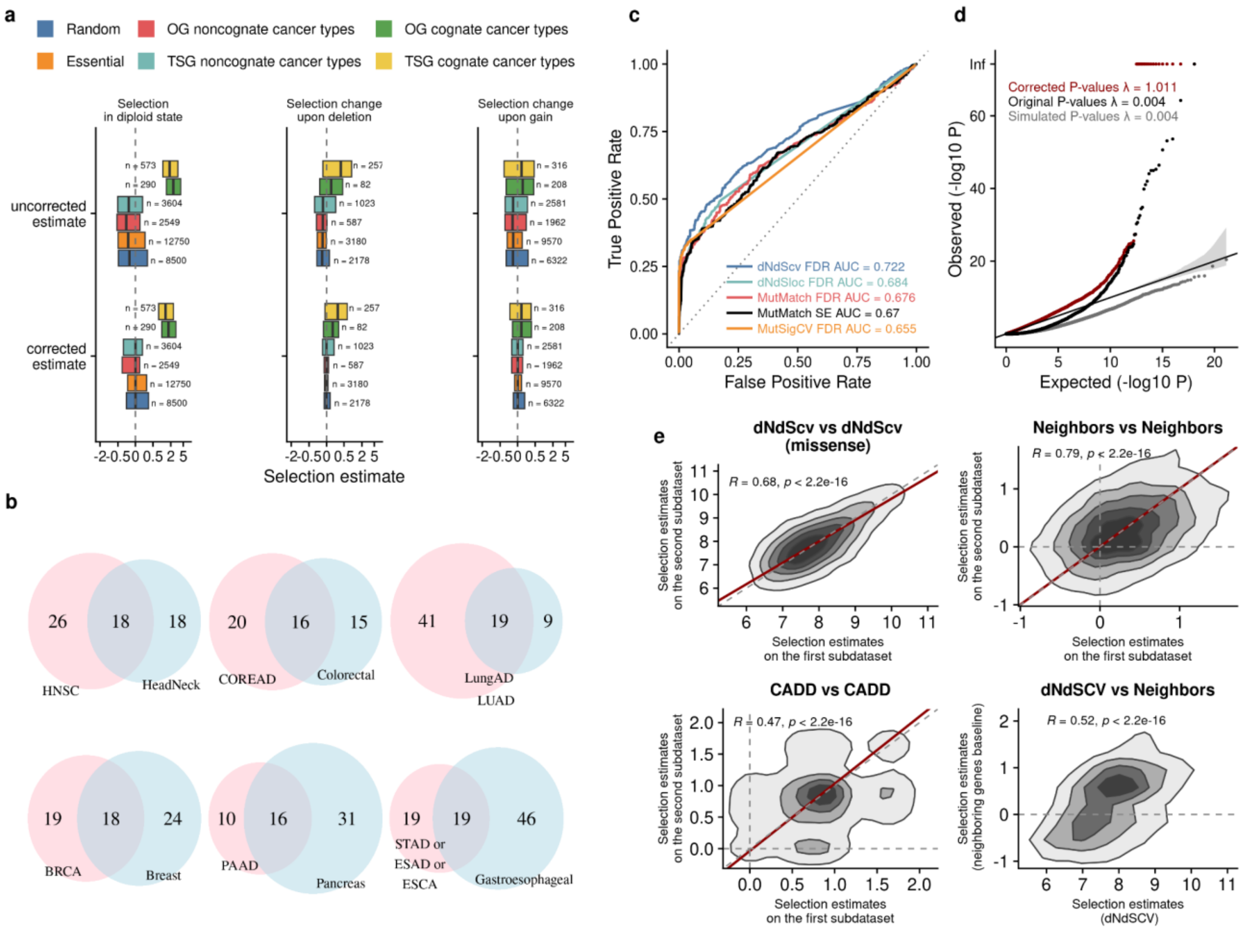

Supplementary Figure S2. Correction procedure removes a bias from selection estimates and overlap between annotation of driver gene-tumor pairs and known driver genes according to MutPanning. a. Selection estimates before and after correction. Estimates for selection on nonsynonymous mutations in a diploid state and a conditional selection in a hemizygous state or cells with copy number gain. Uncorrected estimates tend to be more negative than expected, based on the assumption that random genes should be under neutral selection. Bias-corrected estimates for random genes are centered at zero for all copy number states, as expected. The center line of box plots denotes medians of data points and the box hinges correspond to the 25th and 75th percentiles, hiskers extend to 1.5× IQR from the hinges. **b.** Overlap between selected genes according to the MutPanning method (in blue) and cognate genes defined in this study (in pink) for several cancer types. **c.** AUC based on CGC genes for MutMatch, dNdScv, dNdSloc, and MutSigCV methods. FDR - False Discovery Rate, SE - Selection Estimate ($\log_2$ fold-enrichment in mutation rate). For MutSigCV and dNdScv, q-values from one-tailed p-values were used as a measure of positive selection, while MutMatch both selection estimates or one-tailed q-values were utilized. The analysis benchmarks against the Cancer Gene Census (CGC) to evaluate the performance of each method in distinguishing CGC-annotated driver genes in cognate cancer types (using the MutPanning annotation) from a background of random genes in same cancer types. For all methods mutations from the TCGA MC3 project were used. **d.** Correction of original p-values using the simulated (generated from randomized data) p-values as a null distribution leads to improvement of the inflation factor λ. P-values are shown for random genes (excluding essential genes and genes found in MutPanning or CGC lists of cancer genes). **e.** The correlation of effect size estimates between methodologies or between the same method on orthogonal subset of the data. 2D density plots of distributions are shown, with the dark red lines representing the orthogonal regression line. The MutMatch method

shows a moderate correlation with dNdScv (Pearson's R = 0.52) and a stronger self-correlation (R = 0.79). dNdScv shows a moderate self-correlation is with R = 0.68. Low-impact mutation sites have a weaker correlation (R = 0.47).

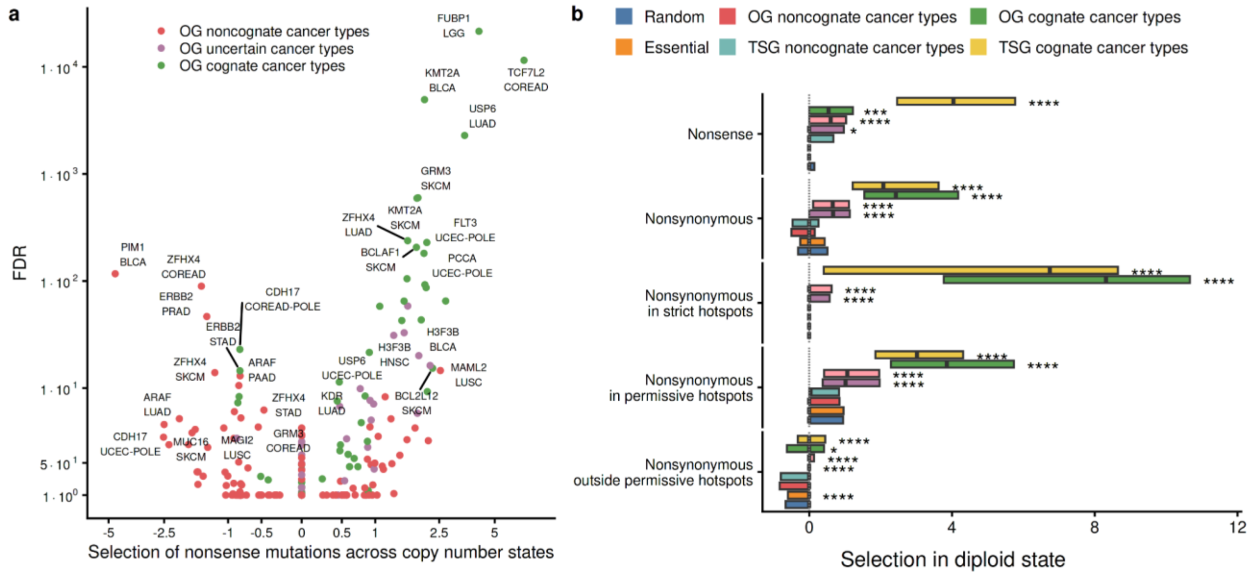

Supplementary Figure S3. Selection estimates for diploid state show signatures of positive and negative selection in oncogenes. a. Selection of nonsense mutations across copy number states for oncogenes. **b.** Selection in diploid state. Debiased selection estimates for the neutral copy number status of a gene obtained on the discovery cohort. The center line of box plots denotes medians of data points and the box hinges correspond to the 25th and 75th percentiles. One data point corresponds to one gene-tumor combination. P-values are by Mann-Whitney U-test, two-tailed. Asterisks indicate the level of significance of the difference between gene groups and a set of random genes for each set of mutations separately: * for FDR ≤ 5%, ** for FDR ≤ 1%, *** for FDR ≤ 0.1%, **** for FDR ≤ 0.01%. The nonsynonymous mutations are the missense and the nonsense mutations considered together. The "Random" genes group is a control that excludes known driver genes and essential genes.

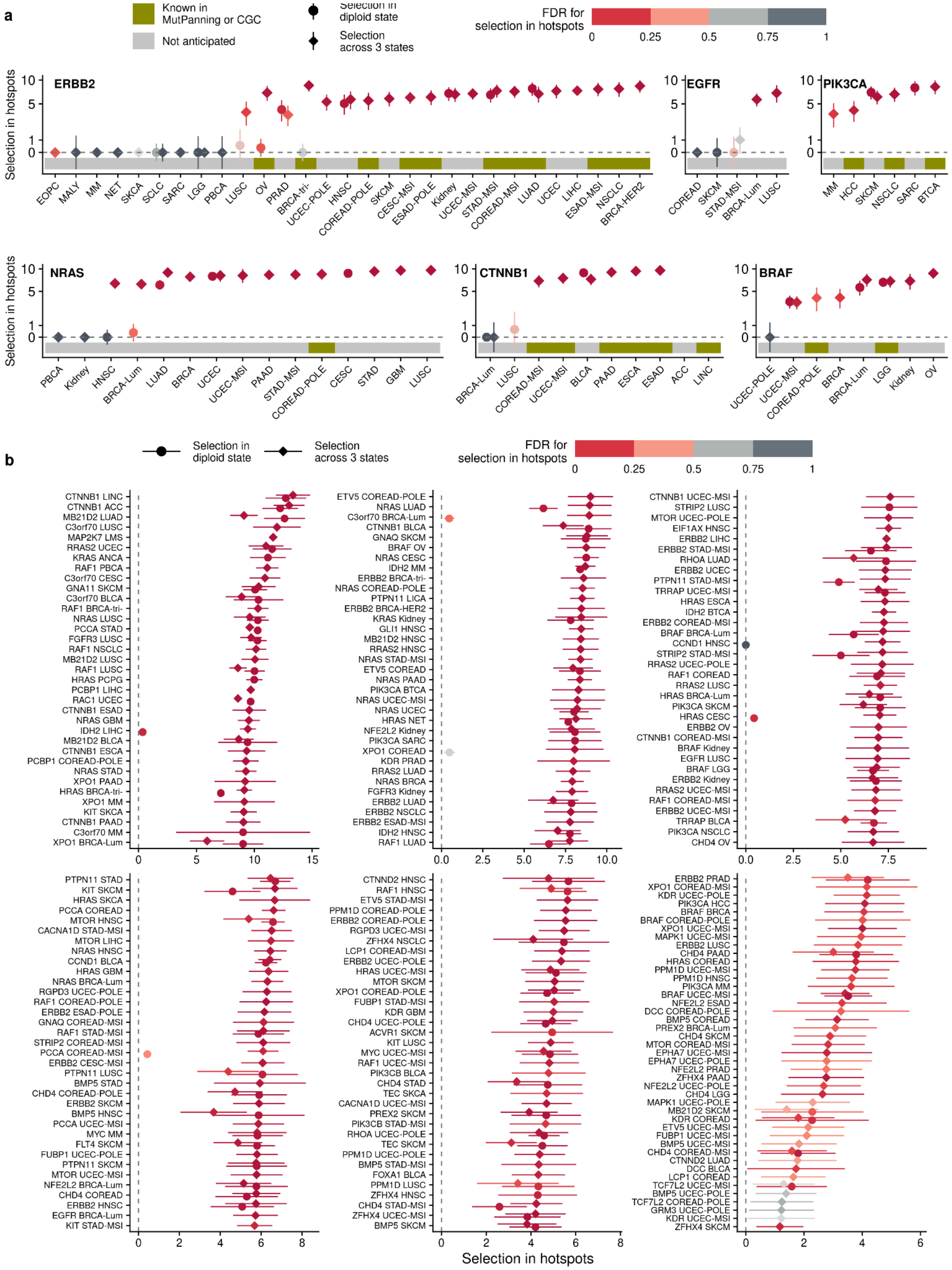

Supplementary Figure S4. Selection in hotspots helps identify new cognate gene-tumor type combinations. a. Selection of *ERBB2*, EGFR, NRAS, *CTNNB1*, *BRAF* and *PIK3CA* genes in noncognate and uncertain cancer types in stringent hotspots. Cancer types where a gene is known to be a driver according to MutPanning or CGC annotations are marked. Error bars show 95% CI. **b.** Hotspot selection in OGs in noncognate cancer types. Only

gene-tumor type pairs with at least one non-zero selection estimate in hotspots (in diploid state or across copy number states) are shown. Error bars show 95% CI.

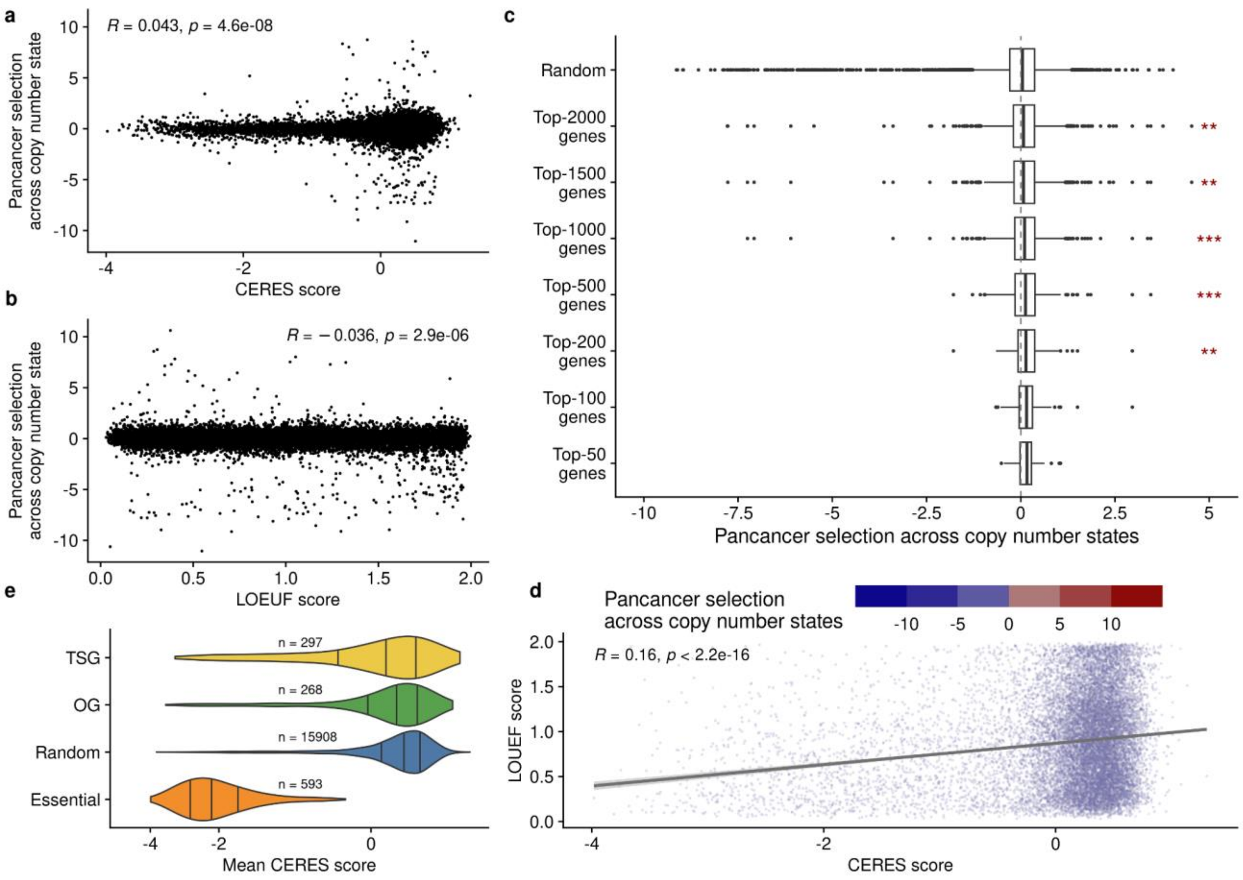

Supplementary Figure S5. Whole-genome analysis of pan-cancer selection. a-b. Somatic cell lines derived and population essentiality scores do not correlate with somatic selection estimated in tumors. Lower values of CERES and LOEUF scores correlate with a higher chance of being essential in cell-line knockout experiments or with haploinsufficiency at the population level. We observe very weak correlation between these essentiality scores and estimates of selection derived with the MutMatch method (across copy number states, pan-cancer analysis) across all genes (Pearson's R=0.043 for CERES score and -0.036 for LOEUF). **c.** The most essential genes according to the LOEUF scores are more positively selected than a set of random genes. The center line of box plots denotes medians of data points and the box hinges correspond to the 25th and 75th percentiles, whiskers extend to 1.5× IQR from the hinges. Asterisks indicate the level of significance of the difference between gene groups and a set of random genes for each set of mutations separately: '*' for FDR ≤ 5%, '**' for FDR ≤ 1%, '***' for FDR ≤ 0.1%, '****' for FDR ≤ 0.01%. **d**. LOEUF and CERES scores are weakly correlated (R=0.16). **e**. CERES scores distribution for OGs, TSGs, essential genes and control set of random genes, where lower scores correspond to higher cell essentiality in CRISPR–Cas9 genetic screens.The center line of violin plots denotes the medians of data points, and the 25th and 75th percentiles are marked within the plot. The width of the plot at different values represents the density of the data points, with wider sections indicating higher densities.

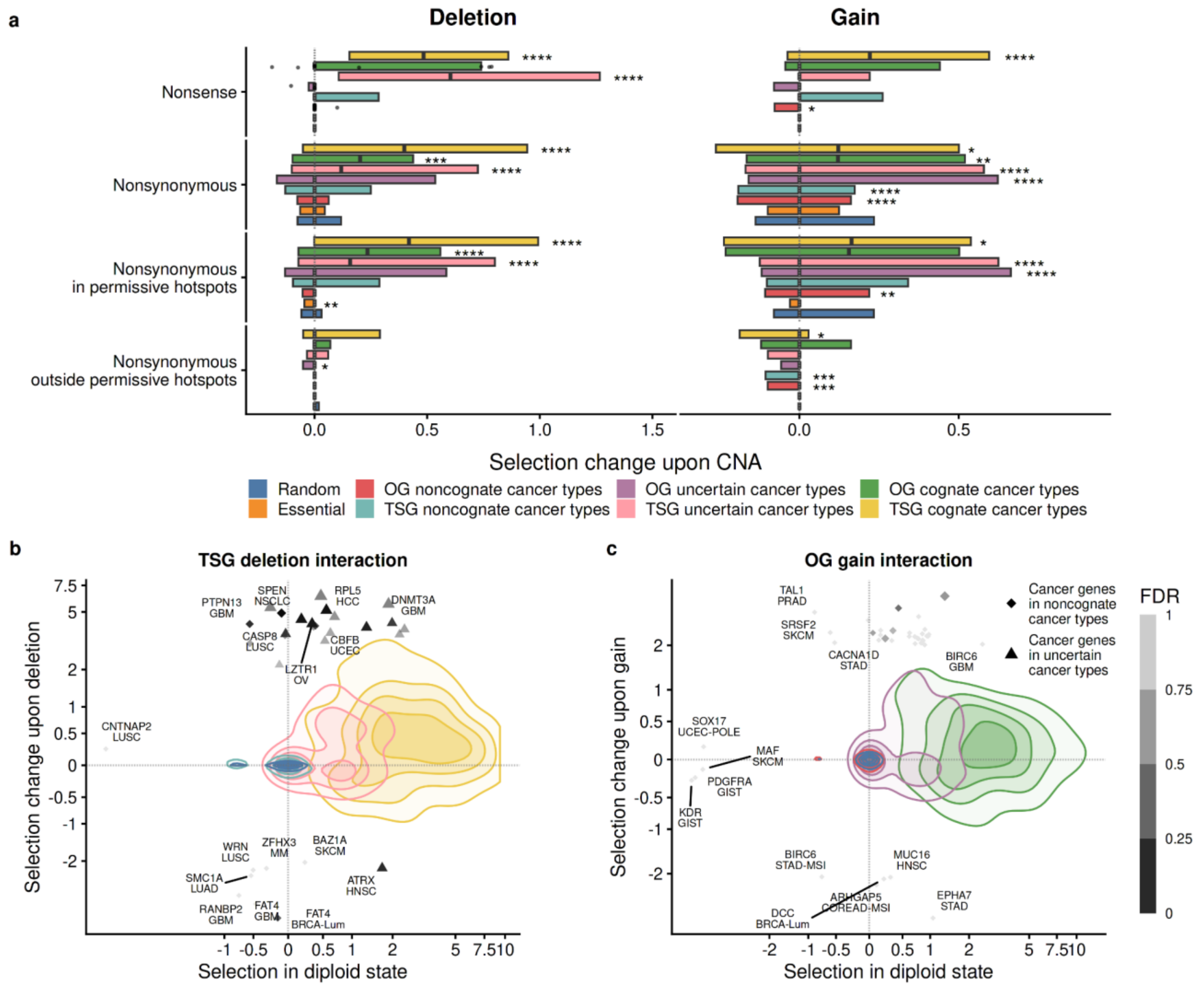

Supplementary Figure S6. Noncognate and uncertain gene-tumor type combinations selected as two-hit genes. a. The change of selection strength between samples where genes are in the diploid state and where a gene copy was lost or gained, estimated using neighboring genes as a mutational rate baseline. Visualization as in **Fig. 3a**, with inclusion of uncertain OGs and TSGs. The center line of box plots denotes medians of data points and the box hinges correspond to the 25th and 75th percentiles. **b, c.** Selection effects of nonsynonymous mutations in cancer genes in noncognate and uncertain cancer types: **(b)** TSGs **(c)** OGs. Genes with the strongest selection change in samples with deletion or gain are labelled. Visualization as in **Fig. 3b-c.**

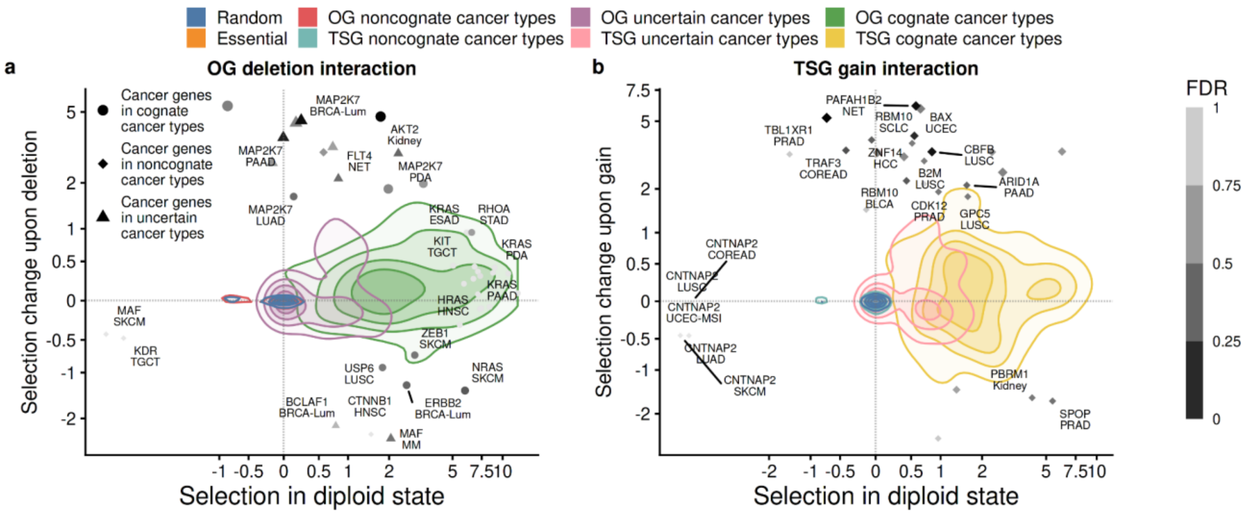

Supplementary Figure S7. Noncognate, cognate and uncertain gene-tumor combinations selected as two-hit genes. Selection effects of nonsynonymous mutations in cancer genes in noncognate cancer types: **(A)** OGs **(B)** TSGs. Genes with the strongest selection change in samples with deletion or gain are labelled.Visualization as in Fig. 4b-c.

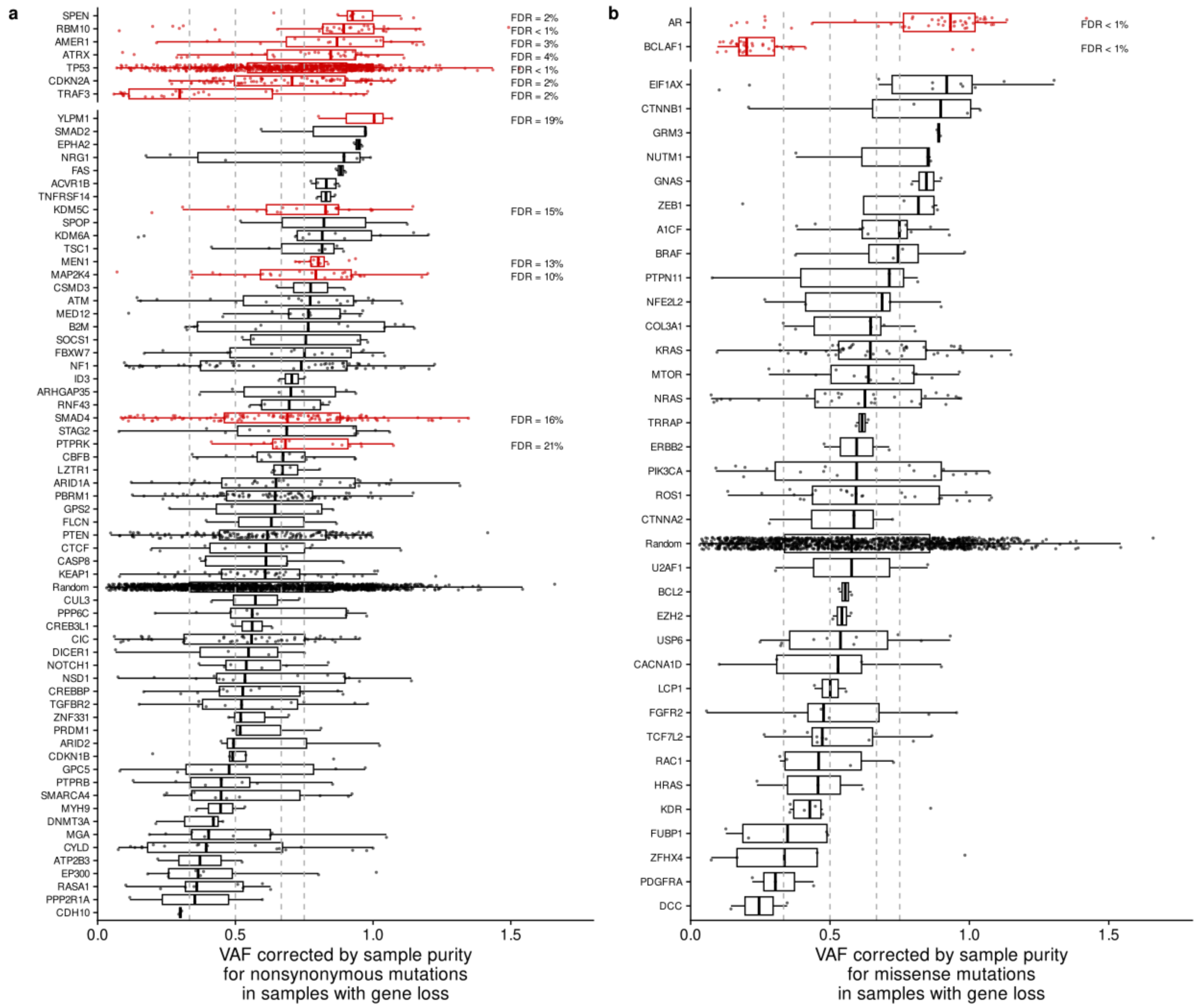

Supplementary Figure S8. Mutation frequencies in samples with a gene loss. a. Variant allele frequencies corrected by the sample purity for TSGs across cognate cancer types (one data point corresponds to one nonsynonymous mutation). The top panel showcases genes where the FDR $\leq$ 5%, while the bottom panel includes all other genes.Genes with FDR $\leq$ 25% are highlighted. The center line of box plots denotes medians of data points and the box hinges correspond to the 25th and 75th percentiles, whiskers extend to 1.5× IQR from the hinges. **b.** Variant allele frequencies corrected by the sample purity for OGs across cognate cancer types (one data point corresponds to one missense mutation). The top panel showcases genes where the FDR $\leq$ 5%, while the bottom panel includes all other genes.Genes with FDR $\leq$ 25% are highlighted. The center line of box plots denotes medians of data points and the box hinges correspond to the 25th and 75th percentiles, whiskers extend to 1.5× IQR from the hinges.

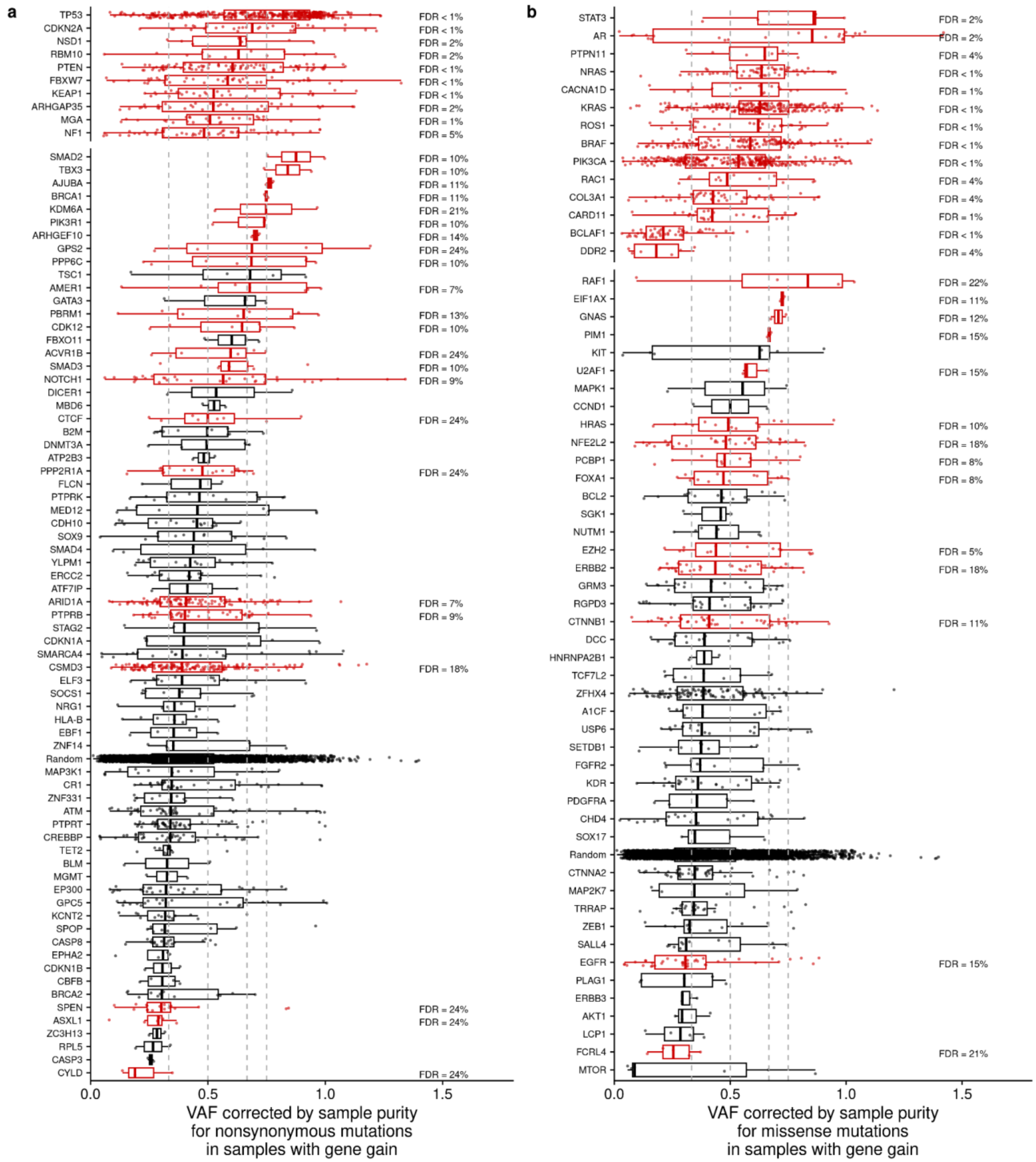

Supplementary Figure S9. **Mutation frequencies in samples with a gene copy number gain. a.** Variant allele frequencies corrected by the sample purity for TSGs across cognate cancer types (one data point corresponds to one nonsynonymous mutation). The top panel showcases genes where the FDR ≤ 5%, while the bottom panel includes all other genes.Genes with FDR ≤ 25% are highlighted. The center line of box plots denotes medians of data points and the box hinges correspond to the 25th and 75th percentiles, whiskers extend to 1.5× IQR from the hinges. **b.** Variant allele frequencies corrected by the sample purity for OGs across cognate cancer types (one data point corresponds to one missense mutation). The top panel showcases genes where the FDR ≤ 5%, while the bottom panel includes all

other genes. Genes with FDR ≤ 25% are highlighted. The center line of box plots denotes medians of data points and the box hinges correspond to the 25th and 75th percentiles, whiskers extend to 1.5× IQR from the hinges.

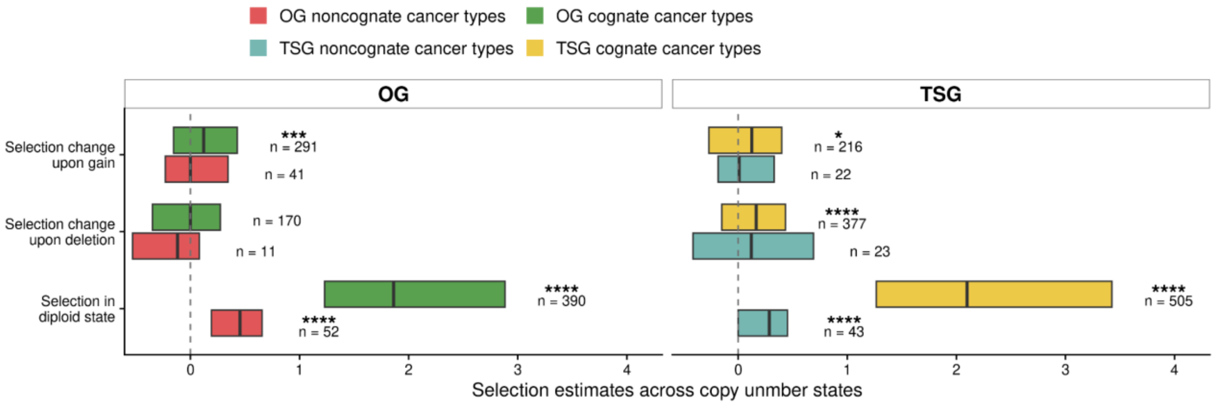

Supplementary Figure S10. Selection on nonsynonymous mutations in diploid state and conditional selection upon CNA events in GENIE study. Debiased regression coefficient ω (estimation of selection pressure in the diploid state and debiased regression coefficients δ on the interaction term between the selection variable t and copy number variable c, for gene deletions, and copy gains. One data point corresponds to one gene-tumor type combination. The center line of box plots denotes medians of data points and the box hinges correspond to the 25th and 75th percentiles. Adjusted p-values (one-sample Mann-Whitney test against zero) are shown for FDR ≤ 25%: ‘*’ for FDR ≤ 5%, ‘**’ for FDR ≤ 1%, ‘***’ for FDR ≤ 0.1%, ‘****’ for FDR ≤ 0.01%.

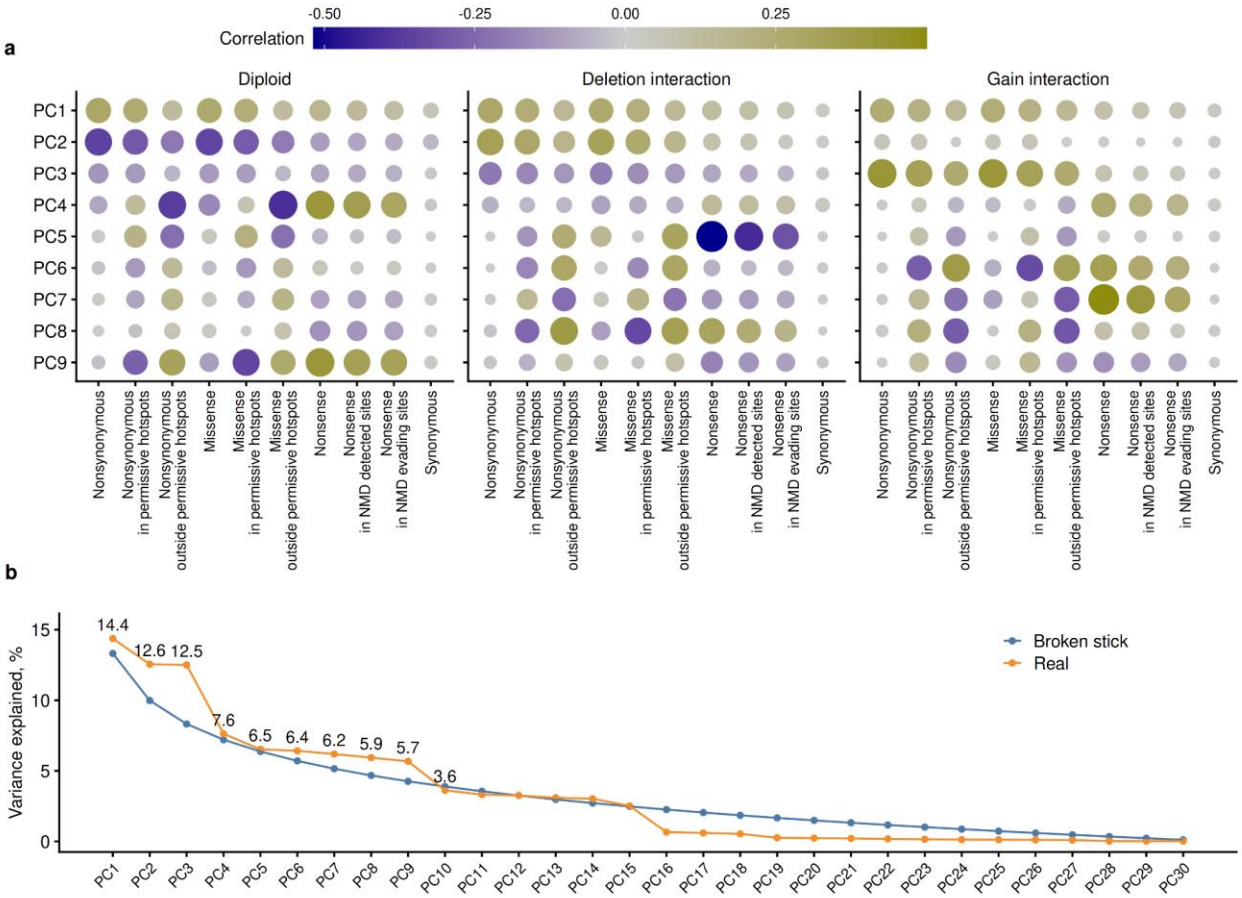

Supplementary Figure S11. PC analysis of selection in different copy number states. a. Loadings (correlations with features) of nine first principle components (prior to rotation). **b.** Proportion of variance explained by the derived principle components (orange) compared to the expected explained variance. Three first PCs were found significant (broken stick test).

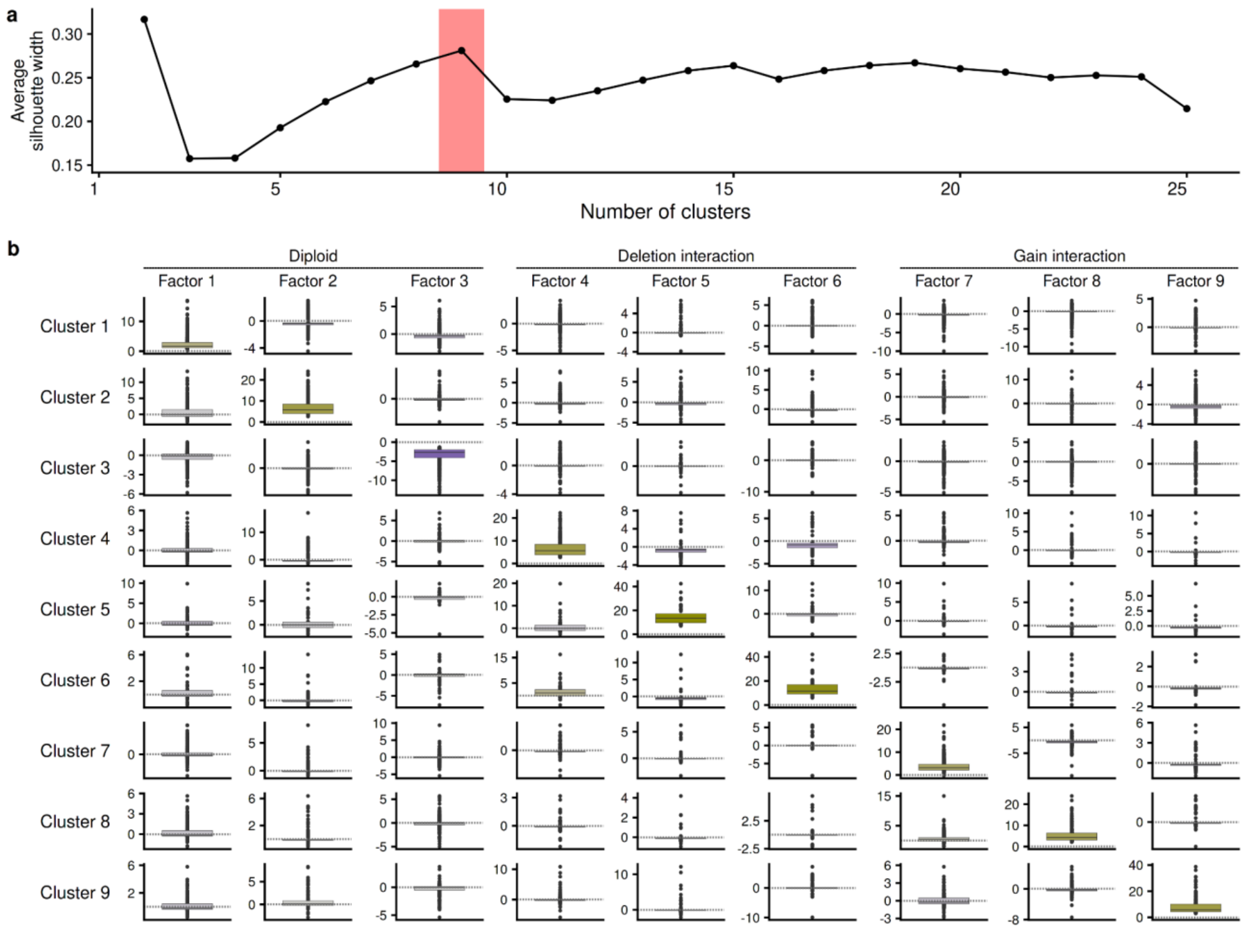

Supplementary Figure S12. Average factor scores for each cluster. a. Average silhouette index width for each number of clusters. 9 clusters were chosen for the final analysis**. b.** Factor (rotated PCs) scores for each cluster of gene-tumor type pairs. One data point represents a gene-tumor type pair. The center line of box plots denotes medians of data points and the box hinges correspond to the 25th and 75th percentiles.

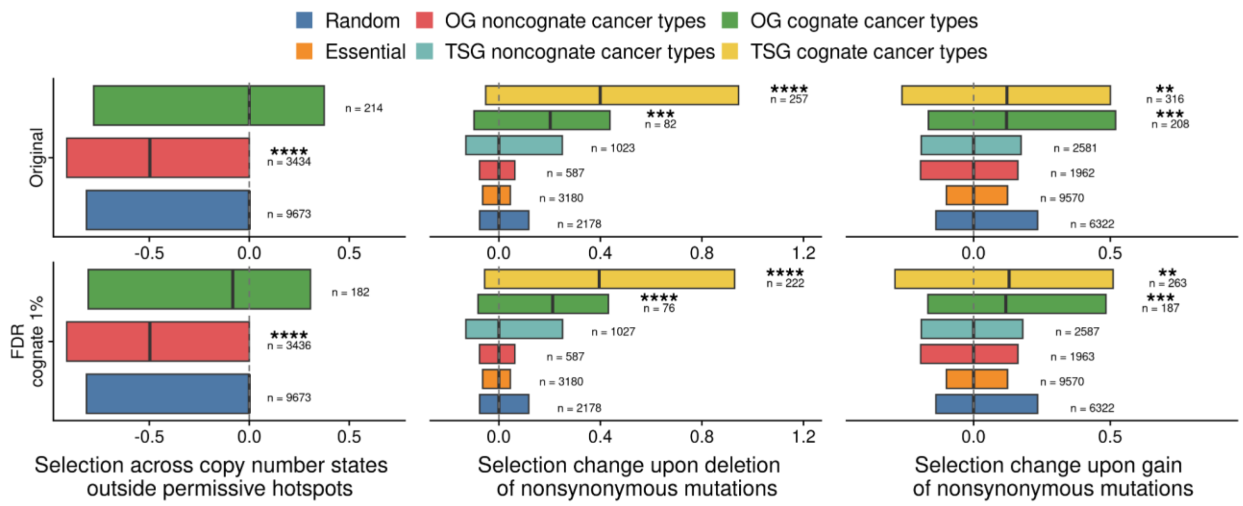

Supplementary Figure S13. The main findings are replicating with different annotations of cognate gene-cancer type pairs using 1% FDR. Strength of selection across all copy number states outside permissive hotspots and the change of selection strength between samples where genes are in the diploid state and where a gene copy was lost or gained, estimated using neighboring genes as a mutational rate baseline. The center line of box plots denotes medians of data points and the box hinges correspond to the 25th and 75th percentiles. Asterisks indicate the level of significance of the difference between gene groups and a set of random genes for each set of mutations separately: * for FDR ≤ 25%, ** for FDR ≤ 10%, *** for FDR ≤ 1%, **** for FDR ≤ 0.1%. One-tailed Mann-Whitney U-test was used.

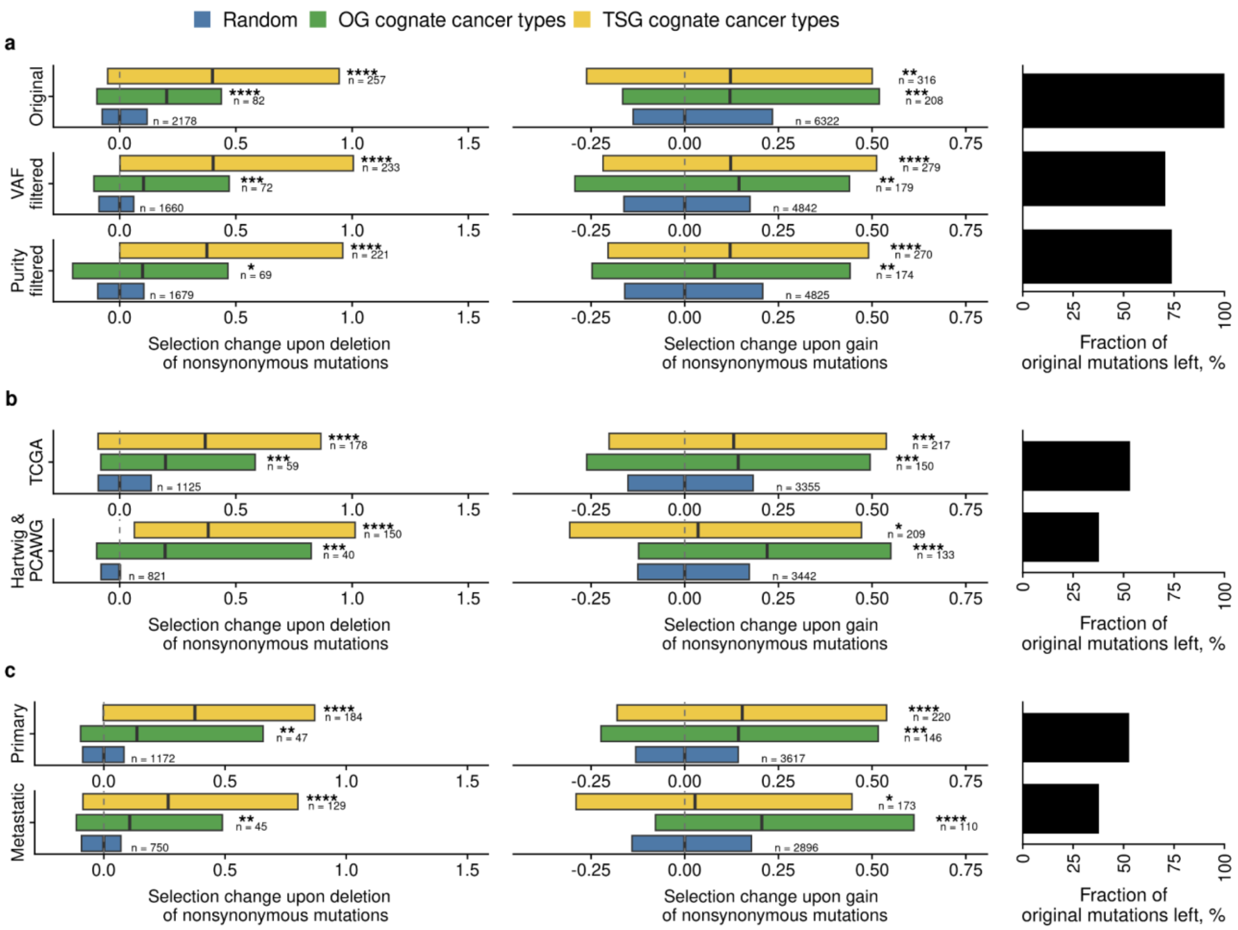

Supplementary Figure S14. The main findings are replicating after filtering mutations with different criteria. Strength of selection across all copy number states outside permissive hotspots and the change of selection strength between samples where genes are in the diploid state and where a gene copy was lost or gained, estimated using neighboring genes as a mutational rate baseline. When a boxplot is generated using no more than 25 data points, each individual data point is displayed. **a.** Filtering to exclude subclonal mutations (those with purity-adjusted VAF<0.25; middle row) and filtering to exclude low-purity samples (<40% estimated purity, bottom row), compared to original data (no filtering, top row). **b.** Separating by cohort and sequencing method, with TCGA WES in top row, and pooled together HMF (Hartwig) and PCAWG WGS in bottom row. **c.** Separating by primary versus metastatic tumors (we note the latter is very enriched with tumor samples from HMF). In all panels, the center line of box plots denotes medians of data points and the box hinges correspond to the 25th and 75th percentiles. Asterisks indicate the level of significance of the difference between gene groups and a set of random genes for each set of mutations separately: * for FDR ≤ 25%, ** for FDR ≤ 10%, *** for FDR ≤ 1%, **** for FDR ≤ 0.1%. One-tailed Mann-Whitney U-test was used.

Supplementary Note 1 for Besedina and Supek (2024) "Copy number losses of oncogenes and gains of tumor suppressor genes generate common driver mutations".

Elucidating cancer type spectrum of driver genes by focussing on known hotspot loci

Some oncogenes-tissue combinations were apparently "noncognate", meaning the positive selection signal was not identified as significant in the overall analysis. However, selection thereon was more evident when analyzing only known hotspot loci; these are shown in **Fig. 2c** (using the stringent hotspot definition) and were identified in the hotspot analysis across copy number states.

Here we discuss two interesting examples involving the *EGFR* gene and *ERBB2* genes. There is apparent selection on hotspot-located mutations in *EGFR* in the luminal subtypes of breast cancer (BRCA) (log_2 fold-enrichment=5.72 across all three copy number states; p=$2.45*10^{-4}$, FDR=$6.87*10^{-3}$). *EGFR* is considered a lung adenocarcinoma and brain cancer driver gene [1,2] and indeed in our analysis *EGFR* was identified as cognate only in GBM, LGG, LUAD and LUSC (at FDR ≤ 2%, **Fig. 2f)**. Given the crucial roles of the closely related gene *ERBB2* (*HER2*) in breast cancer, where *ERBB2* is often amplified and/or overexpressed, a role for *EGFR* alterations would not be unexpected. In breast cancers, *EGFR* is recognized to be amplified and overexpressed [3], and examples of somatic mutations in *EGFR* were discussed in context of druggability [3], however the driver status of *EGFR* mutations in breast cancers was not ascertained in a large-scale genomic analysis [4]. In the literature, a driver role for EGFR was considered for the triple-negative subtype [5,6] and here we provide evidence that *EGFR* would also be a mutation-activated driver gene in the luminal breast cancer subtypes (**Fig. 2c-f**). Next, the amplification-activated oncogene *ERBB2* in several cancer types was significant in this test, where we found selected hotspot point mutations in liver, uterine, pancreas, kidney, melanoma, head-and-neck and prostate cancer (FDR≤25%, at **Fig. 2f**), while *ERBB2* was not marked as cognate in these cancer types (according to our annotation based on across-CNA states selection effects) nor according to MutPanning or Cancer Gene Census (CGC) annotations [1,2].

Further examples of genes where tissue-specificity signal of positive selection was clarified in hotspots include major OGs in noncognate cancer types, prominently *KRAS, BRAF, NRAS, CTNNB1* and *PIK3CA* (**Fig. 2c**). For instance, we find *KRAS* hotspot mutations significant in melanoma, a cancer type where *BRAF* and *NRAS* are well-known driver genes, but the databases Cancer Gene Census and MutPanning do not list *KRAS* as a melanoma driver. Prior literature cites *KRAS* mutations as relevant for some special cases of melanoma tumors, such as brain metastases [7] or genital tract melanoma [8]. We find *NRAS* mutations in hotspots to be selected in lung squamous cell cancer, glioblastoma, stomach, lung adenocarcinoma, cervical cancer, uterus cancer and other cancer types significantly (Supplementary **Fig. 4a**).

The full complement of genes (**Supplementary Fig. S4b)** also consists of various other genes including *CHD4, XPO1, LCP1, FGFR3,* PTPN11, *RAF1* and others. Overall, 110 OG-

cancer type pairs were significant in the hotspot-enrichment analysis using strict or permissive set of hotspots at FDR ≤ 25% but far from significant (FDR ≥ 50% i.e. "noncognate") in the general enrichment of mutations. Similarly, 118 TSG-cancer type pairs were significant in the hotspot test only (FDR ≤ 25%) but were not identified in the general enrichment analysis (FDR ≥ 50% i.e. "noncognate"). Thus, focussing on hotspot regions enriches for positive selection signal, allowing to identify with higher statistical power which driver genes are relevant to which cancer types. Of note, this is related to but distinct from methods that search for significant clustering of mutations, thus defining hotspots *de novo* and identifying new cancer genes [9–11]. The approach described herein relies on known hotspots and thus is not able to identify new cancer genes, but is able to better characterize the cancer type spectrum of known driver genes.

Essential genes show modest signatures of negative selection in somatic cells

In addition to oncogenes, we further considered a set of known cell essential genes (CEG2 set, derived from cell line genetic screening data [12]), analyzed selection in the diploid CNA state on nonsynonymous mutations in a pan-cancer analysis across 13 major cancer types (those with the largest numbers of mutations in our data): BLCA, BRCA-Lum, COREAD, ESAD, HNSC, Kidney, LGG, LUAD, LUSC, MM, PAAD, PRAD and SKCM). Our results show that essential genes are indeed negatively selected both in the pan-cancer analysis in diploid state and across copy number states (for the latter Mann-Whitney $p = 3.85 \times 10^{-7}$, median of random genes = 0.045 and median of CEG2 genes = -0.062; for comparison, median of all OGs = 0.24 and for TSGs = 0.27 in this test that does not distinguish cognate and noncognate cancer types for OG and TSG). Regarding establishing tissue-specificity of these negative selection signatures, given the overall subtle effect sizes, there was not enough power in the dataset to detect the significance in each cancer type separately. The known essentiality metrics correlated only modestly with our negative (purifying) selection estimates across the cancer genomes: CERES score (gene essentiality across cultured cancer cell lines, determined in CRISPR screening experiments [13]) at R = 0.043, and LOUEF population genomic score at R =-0.036 **(Supplementary Fig. S5a-b**). Nonetheless, this CERES score prioritizing genes in cancer cell line genetic screens [12] was more positively correlated with our estimates of purifying selection in cancer genomes, than was the LOEUF score prioritizing genes with organism-level constraint on germline variants [14] (measuring a dearth of germline LoF variants in the population over an expectation), which was anticorrelated (**Supplementary Fig. S5a,c**). The modest correlations notwithstanding, we interpret this to support that some cancer cell-specific negative selection signal, as captured in CERES genetic screening scores, was observed in purifying selection signatures from tumor genomes. As a side note, the two scores (CERES and LOEUF) only weakly correlated at R = 0.16 (**Supplementary Fig. S5d**) suggesting they indeed capture different signals of gene essentiality: cancer cell specific (CERES) versus organism-level (LOEUF). Overall, our results suggest that signatures of negative selection in cancer on point mutations are extant but very subtle, and do not currently allow prioritizing individual genes by essentiality in different cancer types however with much increased sample sizes they may do so.

On a related note, we also compared the distributions of CERES (cancer cell line genetic screening) essentiality scores in different groups of cancer genes, contrasting with random genes (**Fig. 2e, Supplementary Fig. S5e**). Cancer genes were modestly more essential by this criterion (**Supplementary Fig. S5e**): $Q_1 = -0.23$ and median = 0.21 for TSGs, and $Q_1 = -0.0028$ and median = 0.27 for OGs, compared to Q_1=0.11 and median=0.32 for the random genes). A similar result was seen with the population-level LOEUF score (**Fig. 2e**): 68% and 67.5% of OGs and TSGs, respectively, had the LOEUF scores from the lowest 3 deciles (thus the expectation at random would be ~30%). This suggests that functions of cancer genes, considered as a set, tend to be more essential at the level of the cell (CERES) and also at the organismal level (LOEUF). This result is consistent with the observed negative selection on mutations in oncogenes in cancer genomes, as we reported above, and suggests an intriguing possibility that some TSGs could be essential in cancer cells.

SUPPLEMENTARY REFERENCES

1. Dietlein, F. *et al.* Identification of cancer driver genes based on nucleotide context. *Nat. Genet.* **52**, 208–218 (2020).
2. Futreal, P. A. *et al.* A census of human cancer genes. *Nat. Rev. Cancer* **4**, 177–183 (2004).
3. The Cancer Genome Atlas Network. Comprehensive molecular portraits of human breast tumours. *Nature* **490**, 61–70 (2012).
4. Bailey, M. H. *et al.* Comprehensive Characterization of Cancer Driver Genes and Mutations. *Cell* **174**, 1034–1035 (2018).
5. Park, H. S. *et al.* High EGFR gene copy number predicts poor outcome in triple-negative breast cancer. *Mod. Pathol.* **27**, 1212–1222 (2014).
6. Teng, Y. H.-F. *et al.* Mutations in the epidermal growth factor receptor (EGFR) gene in triple negative breast cancer: possible implications for targeted therapy. *Breast Cancer Res.* **13**, R35 (2011).
7. Rabbie, R. *et al.* The mutational landscape of melanoma brain metastases presenting as the first visceral site of recurrence. *Br. J. Cancer* **124**, 156–160 (2021).
8. Cai, Y., Ke, L., Zhang, W., Lu, J. & Chen, Y. Recurrent KRAS, KIT and SF3B1 mutations in melanoma of the female genital tract. *BMC Cancer* **21**, 677 (2021).
9. Arnedo-Pac, C., Mularoni, L., Muiños, F., Gonzalez-Perez, A. & Lopez-Bigas, N. OncodriveCLUSTL: A sequence-based clustering method to identify cancer drivers. *Bioinformatics* **35**, 4788–4790 (2019).
10. Araya, C. L. *et al.* Identification of significantly mutated regions across cancer types highlights a rich landscape of functional molecular alterations. *Nat. Genet.* **48**, 117–125 (2016).
11. Porta-Pardo, E. *et al.* Comparison of algorithms for the detection of cancer drivers at subgene resolution. *Nat. Methods* **14**, 782–788 (2017).
12. Hart, T. *et al.* Evaluation and design of genome-wide CRISPR/SpCas9 knockout screens. *G3 Genes Genomes Genet.* **7**, 2719–2727 (2017).
13. Meyers, R. M. *et al.* Computational correction of copy number effect improves specificity of CRISPR-Cas9 essentiality screens in cancer cells. *Nat. Genet.* **49**, 1779–1784 (2017).
14. Cacheiro, P. *et al.* Human and mouse essentiality screens as a resource for disease gene discovery. *Nat. Commun.* **11**, (2020).
