# Supplementary material for: Copy number losses of oncogenes and gains of tumor suppressor genes generate common driver mutations
Source: Nat Commun. 2024 Jul 20;15:6139. doi: 10.1038/s41467-024-50552-1 (PMC11271286; doi:10.1038/s41467-024-50552-1)
Supplement: Supplementary file 3 — Description of Additional Supplementary Files [file 41467_2024_50552_MOESM3_ESM.pdf]

Description of Additional supplementary files

Supplementary Data S1: Selection estimates across copy number states

Supplementary Data S2: Selection estimates in diploid state

Supplementary Data S3: Selection change associated with the deletion of the gene.

Supplementary Data S4: Selection change associated with the gain of the gene.

Supplementary Data S5: Clusters of gene-tumor type pairs

Supplementary Data S6: Cancer types with largest amount of data
