# Supplementary material for: Copy number losses of oncogenes and gains of tumor suppressor genes generate common driver mutations
Source: Nat Commun. 2024 Jul 20;15:6139. doi: 10.1038/s41467-024-50552-1 (PMC11271286; doi:10.1038/s41467-024-50552-1)
Supplement: Supplementary file 10 — Reporting summary [file 41467_2024_50552_MOESM10_ESM.pdf]

nature portfolio

Corresponding author(s): Fran Supek

Last updated by author(s): June 19, 2024

Reporting Summary

Nature Portfolio wishes to improve the reproducibility of the work that we publish. This form provides structure for consistency and transparency in reporting. For further information on Nature Portfolio policies, see our Editorial Policies and the Editorial Policy Checklist.

Statistics

For all statistical analyses, confirm that the following items are present in the figure legend, table legend, main text, or Methods section.

n/a	Confirmed	
☐	☒	The exact sample size (*n*) for each experimental group/condition, given as a discrete number and unit of measurement
☐	☒	A statement on whether measurements were taken from distinct samples or whether the same sample was measured repeatedly
☐	☒	The statistical test(s) used AND whether they are one- or two-sided<br>*Only common tests should be described solely by name; describe more complex techniques in the Methods section.*
☐	☒	A description of all covariates tested
☐	☒	A description of any assumptions or corrections, such as tests of normality and adjustment for multiple comparisons
☐	☒	A full description of the statistical parameters including central tendency (e.g. means) or other basic estimates (e.g. regression coefficient) AND variation (e.g. standard deviation) or associated estimates of uncertainty (e.g. confidence intervals)
☐	☒	For null hypothesis testing, the test statistic (e.g. *F*, *t*, *r*) with confidence intervals, effect sizes, degrees of freedom and *P* value noted<br>*Give P values as exact values whenever suitable.*
☐	☒	For Bayesian analysis, information on the choice of priors and Markov chain Monte Carlo settings
☒	☐	For hierarchical and complex designs, identification of the appropriate level for tests and full reporting of outcomes
☐	☒	Estimates of effect sizes (e.g. Cohen's *d*, Pearson's *r*), indicating how they were calculated

Our web collection on statistics for biologists contains articles on many of the points above.

Software and code

Policy information about availability of computer code

Data collection	We used previously collected data available from either public databases or other publications as described in Methods.
Data analysis	ANNOVAR was used for functional annotation of somatic mutations. liftOver was employed for converting genomic coordinates.<br>The remaining analyses were carried out using custom code within the R environment (version 3.5 and 3.6). Relevant R packages are data.table, dplyr, ComplexHeatmap, ggplot2, MASS, arm, fastglm, GenomicRanges, BioStrings, rtracklayer, devtools.

For manuscripts utilizing custom algorithms or software that are central to the research but not yet described in published literature, software must be made available to editors and reviewers. We strongly encourage code deposition in a community repository (e.g. GitHub). See the Nature Portfolio guidelines for submitting code & software for further information.

Data

Policy information about availability of data

All manuscripts must include a data availability statement. This statement should provide the following information, where applicable:

- Accession codes, unique identifiers, or web links for publicly available datasets
- A description of any restrictions on data availability
- For clinical datasets or third party data, please ensure that the statement adheres to our policy

The relevant data generated is included in the Supplementary material and can be found at this link: https://figshare.com/articles/dataset/Supplementary_Data/26029309 . Interactive visualizations for selected figures from this publication are available at https://ebesedina.shinyapps.io/

mutmatch_web/. Previously published data and resources used in this work: vcf files and CNV data for TCGA WGS [https://portal.gdc.cancer.gov/], HMF [https://www.hartwigmedicalfoundation.nl/en/], TCGA WES mutation calls from MC3 [https://gdc.cancer.gov/about-data/publications/mc3-2017], TCGA WES CNA calls [https://portal.gdc.cancer.gov/], PCAWG [https://dcc.icgc.org/pcawg/], POG570 [https://www.bcgsc.ca/downloads/POG570/], CPTAC-3 [https://portal.gdc.cancer.gov/], MMRF-COMPASS [https://portal.gdc.cancer.gov], GENIE [https://www.synapse.org/], Project Score CRISPR genetic screening data [https://depmap.org/portal/download/], gnomAD [https://gnomad.broadinstitute.org/downloads], CADD scores [https://krishna.gs.washington.edu/download/CADD/bigWig/], NMDetective [https://figshare.com/articles/dataset/NMDetective/7803398], CRG75 Alignability track [https://hgdownload.soe.ucsc.edu/goldenPath/hg19/database/], CUP bed files [https://github.com/cathaloruaidh/genomeBuildConversion/#2-novel-cup-bed-files], Top-rank expressed transcripts of protein-coding genes [https://tregt.ibms.sinica.edu.tw/index.php#tab6]. Source data are provided with this paper.

Research involving human participants, their data, or biological material

Policy information about studies with human participants or human data. See also policy information about sex, gender (identity/presentation), and sexual orientation and race, ethnicity and racism.

Reporting on sex and gender	Sex and gender are not pertinent to the study.
Reporting on race, ethnicity, or other socially relevant groupings	Ethnicity or other socially relevant groupings are not relevant to the study.
Population characteristics	Study does not address population characteristics.
Recruitment	There was no recruitment; data from existing databases was analyzed.
Ethics oversight	Does not apply.

Note that full information on the approval of the study protocol must also be provided in the manuscript.

Field-specific reporting

Please select the one below that is the best fit for your research. If you are not sure, read the appropriate sections before making your selection.

☒ Life sciences ☐ Behavioural & social sciences ☐ Ecological, evolutionary & environmental sciences

For a reference copy of the document with all sections, see nature.com/documents/nr-reporting-summary-flat.pdf

Life sciences study design

All studies must disclose on these points even when the disclosure is negative.

Sample size	To form the discovery data set for our analyses, we collected from various databases the exomes and genomes of ~23 000 tumor samples with mutations from 117 cancer types or subtypes, of which ~18 000 had both mutation and CNA data available. The number of tumor samples per each tumor type was ranging from 1 to over 1000 for PRAD (prostate adenocarcinoma), BRCA-Lum (a luminal subtype of breast cancer), COREAD (colorectal adenocarcinoma), MM (multiple myeloma), kidney, PAAD (pancreatic adenocarcinoma), and SKCM (skin cutaneous melanoma). The median number of tumor samples per cancer type was 57. The average number of SNV mutations in each cancer type was 28 805, and the median number of mutations was 4 136. For the validation dataset, we downloaded mutation calls for 90 713 tumor samples across 75 cancer types.
Data exclusions	We only focused on gene-tumor type pairs that had at least 2 (3) mutations across all samples where a gene was in a copy number neutral state, and at least 2 (3) mutations in samples where a gene was somatically deleted or gained, for the discovery (and validation) datasets, respectively; these counts were considered separately for each cancer type. For the pan-cancer analysis we required at least 10 mutations to occur in the gene of interest across all cancer types used, and for selection estimates across copy number states we required at least 8 mutations to be present in each regression model.
Replication	Discovery and validation cohorts were considered as independent replicates. Analyses of the validation cohort, and analysis using VAF were used to validate the main findings about selection on cancer genes in the discovery cohort. Main findings discussed in the paper were validated using VAF analysis and the validation cohort.
Randomization	Does not apply; there was no randomization into groups, since this is an observational data analysis.
Blinding	Blinding was not relevant because of the observational rather than interventional nature of this study.

Reporting for specific materials, systems and methods

We require information from authors about some types of materials, experimental systems and methods used in many studies. Here, indicate whether each material, system or method listed is relevant to your study. If you are not sure if a list item applies to your research, read the appropriate section before selecting a response.

Materials & experimental systems

n/a	Involved in the study
☒	☐ Antibodies
☒	☐ Eukaryotic cell lines
☒	☐ Palaeontology and archaeology
☒	☐ Animals and other organisms
☒	☐ Clinical data
☒	☐ Dual use research of concern
☒	☐ Plants

Methods

n/a	Involved in the study
☒	☐ ChIP-seq
☒	☐ Flow cytometry
☒	☐ MRI-based neuroimaging
